# Stochastic anomaly of methylome but persistent SRY hypermethylation in disorder of sex development in canine somatic cell nuclear transfer

**DOI:** 10.1038/srep31088

**Published:** 2016-08-09

**Authors:** Young-Hee Jeong, Hanlin Lu, Chi-Hun Park, Meiyan Li, Huijuan Luo, Joung Joo Kim, Siyang Liu, Kyeong Hee Ko, Shujia Huang, In Sung Hwang, Mi Na Kang, Desheng Gong, Kang Bae Park, Eun Ji Choi, Jung Hyun Park, Yeon Woo Jeong, Changjong Moon, Sang-Hwan Hyun, Nam Hyung Kim, Eui-Bae Jeung, Huanming Yang, Woo Suk Hwang, Fei Gao

**Affiliations:** 1 Sooam Biotech Research Foundation, Seoul 152-904, Korea; 2 BGI-Shenzhen, Shenzhen, China; 3 Agricultural Genomics Institute at Shenzhen, Chinese Academy of Agricultural Sciences, Shenzhen, China; 4 Animal Bioscience and Biotechnology Laboratory, United States Department of Agriculture, Beltsville, MD, 20705, USA; 5 Department of Animal and Avian Sciences, University of Maryland, College Park, MD, 20742, USA; 6 College of Veterinary Medicine, Chonnam National University, Gwangju 500-757, Korea; 7 College of Veterinary Medicine, Chungbuk National University, Cheongju, Chungbuk 361-763, Korea; 8 College of Animal Sciences, Chungbuk National University, Cheongju, Chungbuk 361-763, Korea

## Abstract

Somatic cell nuclear transfer (SCNT) provides an excellent model for studying epigenomic reprogramming during mammalian development. We mapped the whole genome and whole methylome for potential anomalies of mutations or epimutations in SCNT-generated dogs with XY chromosomal sex but complete gonadal dysgenesis, which is classified as 78, XY disorder of sex development (DSD). Whole genome sequencing revealed no potential genomic variations that could explain the pathogenesis of DSD. However, extensive but stochastic anomalies of genome-wide DNA methylation were discovered in these SCNT DSD dogs. Persistent abnormal hypermethylation of the SRY gene was observed together with its down-regulated mRNA and protein expression. Failure of SRY expression due to hypermethylation was further correlated with silencing of a serial of testis determining genes, including SOX9, SF1, SOX8, AMH and DMRT1 in an early embryonic development stage at E34 in the XY^DSD^ gonad, and high activation of the female specific genes, including FOXL2, RSPO1, CYP19A1, WNT4, ERα and ERβ, after one postnatal year in the ovotestis. Our results demonstrate that incomplete demethylation on the *SRY* gene is the driving cause of XY^DSD^ in these XY DSD dogs, indicating a central role of epigenetic regulation in sex determination.

Mammalian sex determination, which is the genetic process by which gonadal primordia are committed to differentiate into either testes or ovaries, is governed by complex molecular networks of gene expression^1^. The products of testis and ovary specific genes antagonize each other and promote testis and ovary development. Disturbance of this equilibrium can lead to disorders of sex development (DSDs)^2^. In humans, one in 4,500 infants is born with DSD, confronting both the parents and associated medical staff with challenging decisions regarding gender assignment, genital surgery and lifelong care^3^,^4^. Improved understanding of the genetic causes of such disorders will lead to refinements in their diagnosis and management.

Most of our current knowledge regarding mammalian sex determination is derived from studies of sex-reversed mice obtained by direct genetic manipulation or natural cases of exceptions to normal gonad development^5^. Previous reports mainly focused on genetic mutations, suggesting that DSD is typically caused by loss-of function mutations in genes required for formation and maintenance of the gonadal primordia, resulting in varying degrees of gonadal dysgenesis. However, a specific molecular diagnosis currently only be made in ~20% of patients with DSDs.

Cloned animals generated by somatic cell nuclear transfer (SCNT) technology provide an alternative model as cases of anomalies in the sex differentiation of animals have reportedly occurred during SCNT procedure for unknown reasons^6^. This evokes questions about early events of epigenetic reprogramming in SCNT when the chromosomal sex is clearly determined in genetic donor cells. Epigenetic regulation of mammalian sex determination has also been revealed as a key mechanism^7^, while incomplete methylation reprogramming in SCNT embryos is actually a common issue^8^.

In the present study, we describe our observations in SRY positive XY DSD with almost complete female phenotypes in dogs generated by SCNT technology. By applying whole-genome and whole-methylome sequencing on three pairs of donor-clone dogs, we revealed persistent hypermethylation of the SRY gene as the main cause of XY DSD, in addition to extensive genome-wide anomaly of methylation, while no genomic variations could explain the XY DSD in our study. Our results clearly indicated the central role of epigenetic regulation on SRY in sex determination, as well as the importance of the epigenomic reprogramming during early embryonic development.

## Results

### Cytogenetic and phenotypic analyses of DSD dogs generated by SCNT

**Figure 1:**
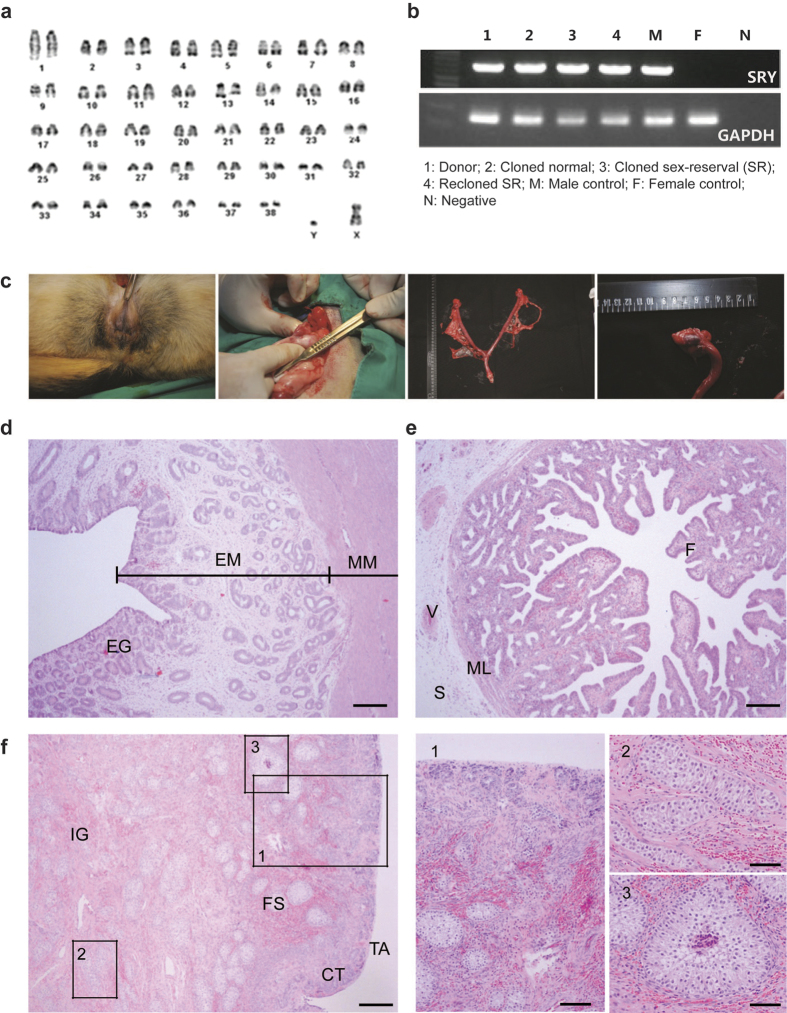
XY^DSD^ phenotype of cloned dog produced by SCNT. (**a**) Karyotyping analysis of the fibroblast of a sex reversed cloned dog (78, XY); (**b**) Agarose gel electrophoresis of SRY-specific PCR product; (**c**) External genitalia of a cloned male (XY genotype, XX phenotype) showing complete gonadal dysgenesis (left) and its gonad (right); (**d–f**) Hematoxylin/eosin (H&E) stained gonad and epididymic sections from adult XY^DSD^ cloned dog. (**d**) Uterine horn. (**e**) Uterine tube. (**f**) Ovary. CT, cortical tubule; EG, endometrial gland; EM, endometrium; F, fold; FS, follicle-like structure; IG, interstitial gland; ML, muscular layer; MM, myometrium; S, serosa; V, vessel. Scale bars, 50 μm (**d–f**), 100 μm (1), 200 μm (2 and 3).

During our canine somatic cell nuclear transfer (SCNT), we observed an unexpectedly high frequency of the distinct congenital anomaly of male-female sex-reversal. Overall, 22.7% (10/44) of cloned dogs belonging to eight different breeds were found to have this anomaly. All cases occurred in males, but instances were not confined to a particular breed or age of genetic donors (Supplemental Table S1). As exemplified in one sex-reversal case (GSF74), genetic analysis of the metaphase of skin fibroblasts indicated a typically normal male diploid genome (78 XY) (Fig. 1a) without deletion of SRY gene sequences (Fig. 1b). However, its external genitalia showed complete gonadal dysgenesis (Fig. 1c) with typical structures of the uterine horn and tube in normal bitches as revealed by histological analyses (Fig. 1c–e). An ovary-like structure was also observed in the location of normal ovaries, but no mature follicles or oocytes were present (Fig. 1f), and there was no significant increase in any hormones (testosterone, estradiol and progesterone), indicating gonadal failure (Supplemental Fig. 1). These results indicated that the internal and external genital structures were female phenotype, but the streak gonads failed to produce sex hormones. Based on these findings, the cloned dog with the XY sex-reversal can be classified as 78, XY disorder of sex development (78, XY^DSD^), complete gonadal dysgenesis^2^,^4^. Furthermore, re-cloning of this 78 XY dog resulted 83.30% of the re-cloned dogs showing female phenotype without restoring their normal sex condition (Supplemental Table S1).

### No genomic variations revealed in the genomes could potentially explain the XY DSD

To infer potential genetic variations that occurred during the SCNT procedure that could lead to abnormal sex-reversal phenotype, we conducted whole genome sequencing (WGS) on DNA extracted from primary fibroblast cells isolated from seven dog samples, including two normal donors, two sex-reversed cloned dogs, one sex-reversed re-cloned dog and a pair of independent replicate samples taken as controls (Supplemental Table 2). For each genome, an average sequencing depth of 27X was used for the following analyses (see the methods section). The published canine genome originated from a female boxer with no reference sequences assembled for the Y chromosome^9^. To assemble the Y chromosome sequences, we first extracted all pair-ended unmapped reads as the candidate reads originated from chromosome Y. After removing the low-frequency k-mers regions that were generally taken as sequencing errors, we constructed a draft assembly for dog chromosome Y (deposited in SRA). To assess the accuracy of the assembly results, we compared our assembled scaffolds with dog Y-chromosomal fragments available in GenBank. We collected a total of 35 specific fragments from the chrY of dog, the length of which was 27,378 bp. The LASTZ sequence alignment program revealed that all fragments were aligned perfectly to the 12 scaffolds, indicating that our assembly results are accurate and reliable (Supplemental Table 3). A 1495 bp sequence (AF107021) containing a single exon of the canine SRY gene reported by Meyers-Wallen *et al*.^10^ was aligned to scaffold 2360, with its open reading frame (256–915bp) mapped to a region within scaffold 2360 (1057–1719 bp) without gaps.

To separate true variations from machine artifacts as a result of the high rate and context-specific nature of sequencing errors in NGS, we sequenced an independent sample twice, then used the results to evaluate sequencing errors and false positive calls. After obtaining the full canine genome, we conducted a stringent analysis to screen SNVs or indels of the clone sample based on comparison with the donor sample by identification of *de novo* somatic mutations (dnSM) and loss of heterozygosity (dnLOH). The results revealed a similar number of SNVs or indels between the clone-donor sample pairs as between the control replicates (Supplemental Table 4). These findings strongly indicated that there were still false positive calls. Therefore, we filtered out around 40–50% of the SNVs and 70% of the indels using RepeatMasker and re-performed the alignment and variation calling on the re-mapped reads based on more strict criteria (see the methods section) (Supplemental Table 4). Furthermore, we integrated three algorithms to infer potential structure variation (SV) (see the methods section), resulting in a limited number of *de novo* SVs between the donor and clone (Supplemental Table 5). However, there were still a similar number of variants called out from the independent replicates. Further annotation indicated that only a few of the variants were located in exonic regions. We finally performed comprehensive validation of these exonic variants (25 SNPs and 1 Indel) by applying a new method combining PCR amplification and high-throughput indexing MiSeq sequencing (Supplemental Table 6). Overall, 19 of the 25 SNPs were successfully recapitulated in one of the clone-control pairs (Supplemental Table 7). However, none of these recapitulated SNPs were overlapped among the three clone-control pairs (Supplemental Fig. 2). Despite these stochastic variants during the SCNT procedure, no SNVs, indels or SVs of the genes relative to normal sex development were identified based on the criteria we set. To further eliminate potential false negative detection and validate these results, we selected eight genes (SRY, SOX9, AMH, WNT4, SF1, WNT1, WT1 and DMRT1), which are key regulators for sex determination^2^, and applied PCR-based high-throughput indexing HiSeq sequencing to validate the results. However, we still could not detect any variants in the exonic region of these genes (Supplemental Tables 6 and 7).

### Stochastic anomaly of genome-wide DNA methylation in DSD cloned dogs

**Figure 2:**
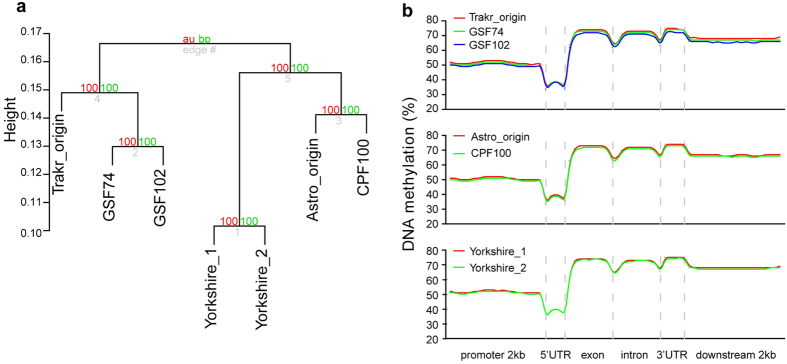
Genome wide DNA methylation patterns of donor and XY^DSD^ cloned dogs. (**a**) Hierarchical clustering of genome-wide CpG methylation in donor and XY^DSD^ cloned dogs using PVclust software. (**b**) Methylation levels (y-axis) of 100-bp intervals around genic regions (x-axis).

Epigenetic abnormalities of cloned animals generated from SCNT technology have previously been observed^8^,^11^,^12^. We next performed whole genome bisulfite sequencing to examine the methylation profiles of the seven samples (Supplemental Table 8). A hierarchical clustering analysis based on the DNA methylation level of CpG sites was performed, which resulted in three distinct branches that each comprised a pair of donor-clone dogs. The difference between a donor and its clone is smaller than the inter-individual difference. Based on the cluster of independent replicate samples as negative controls, dissimilarity between donor and clone is indicated by the cluster height (see methods for “Pvclust” algorithm), which is slightly bigger than the difference between the Trakr clone and reclone (Fig. 2a). However, the average methylation levels of CpG sites in thirteen specific genomic elements, including the genic region (Fig. 2b), CpG island (CGI), CGI shores, simple repeat regions, low complexity region, and LINE region, were not notably different (Supplemental Fig. 3). These results suggested that the differences between the donor-clone sample pairs were not extensive, and were instead restricted to specific loci. Therefore, we further conducted pair-wise comparison of methylation levels across the genomes of the donor-clone sample pairs, revealing about 10,000 differentially methylated regions (DMRs). In contrast, only thirty DMRs were identified between two independent replicates, which was a false positive call (Supplemental Table 9). These results suggested that regional methylation changes can still frequently occur in these DSD dogs, despite the large-scale stability of the methylome.

Because promoter methylation plays a role in gene repression^13^, we focused our analyses on genes containing DMR within their promoter regions. We found more than three hundred promoter DMRs between clones and donors. However, only a small portion of the DMR-containing genes between clones and donors were recurrent among three pairs of donor-clone comparisons, suggesting that these methylation anomalies were mostly stochastic (Supplemental Fig. 4). Furthermore, Gene Ontology enrichment analyses of these promoter-DMR genes (see the methods section) indicated that some subsets of the DMR genes were enriched with GO terms for metabolism or organ development, suggesting potential molecular abnormalities. No GO terms were shared by any two of the three donor-clone sample pairs, again indicating the stochasticity of abnormal methylation reprogramming during SCNT (Supplemental Table 10).

**Figure 3:**
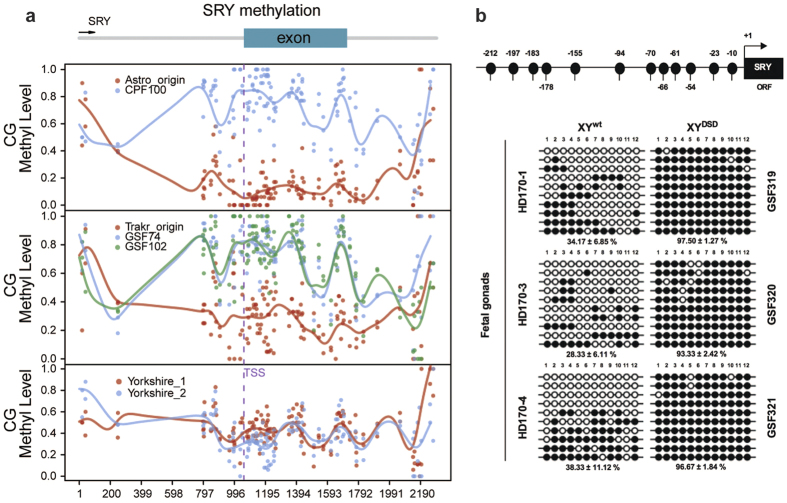
Methylation status of SRY gene in donor/XY^wt^ and XY^DSD^ cloned dogs in E34 fetal gonads. (**a**) Methylation level (y-axis) across SRY gene (x-axis) of skin fibroblast in the donor and XY^DSD^ cloned dogs, data generated from WGBS; (**b**) Promoter methylation status of SRY gene at E34 gonad in the XY^wt^ and XY^DSD^ cloned dogs, using Bisulfite Sequencing PCR (BSP). Each BSP product was subcloned. Ten clones were subjected to nucleotide sequence analysis. Open and filled circles indicate unmethylated and methylated status, respectively.

Sexually dimorphic gene expression has previously been observed in multiple somatic tissues^14^. As a key gene regulation mechanism, we speculated that sexually dimorphic methylation will also be observed in fibroblasts of these dogs. Therefore, we carefully examined the methylation status of the 46 gender-related genes. We found that both the promoter and the gene body of the Y-chromosome-linked gene, *SRY*, consistently displayed significant hypermethylation in the clone samples relative to the donor samples (Fig. 3a), though such divergences were not observed in the other 45 genes (Supplemental Table 11). We further compared the cloned dogs with or without DSD that were cloned from these two donors (Trakr and Astro), revealing much higher methylation level (>90%) of *SRY* gene in DSD than in the normal sex cloned dogs (Supplemental Fig. 5A,B). Moreover, the *SRY* hypermethylation was also observed in two extra cases of DSD cloned dogs that we randomly selected, suggesting it’s a type of recurrent aberrancy in the population (Supplemental Fig. 5C,D).

### Hypermethylation of SRY correlated with male differentiation repression and female phenotypic development in the XY^DSD^ gonad

**Figure 4:**
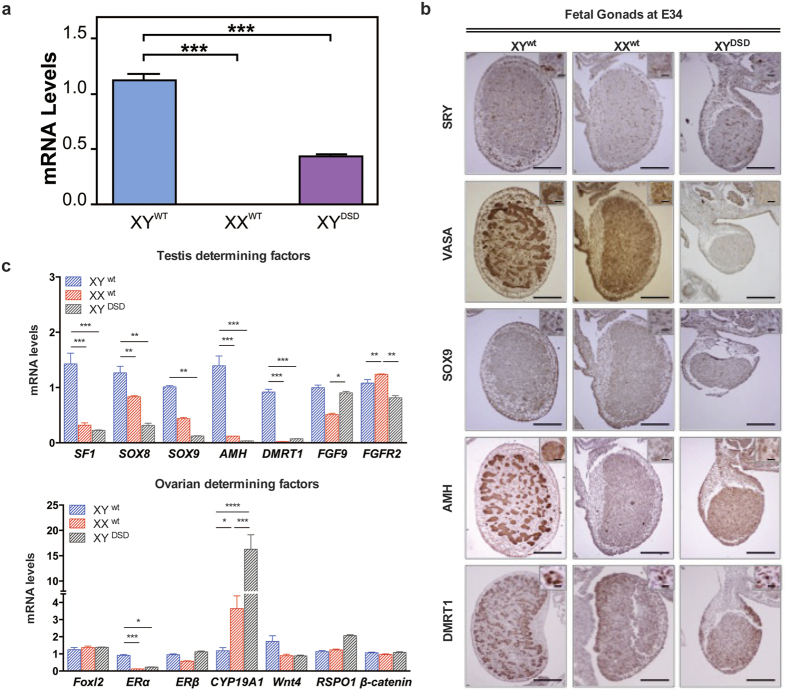
Gene expression of sex-determining genes in E34 fetal gonads of XY^wt^, XX^wt^, and XY^DSD^ cloned dogs. mRNA expression of SRY (**a**) and other (**c**) testis or ovarian determining factors were examined by QPCR. Significance of expression changes is indicated (one-way ANOVA). (**b**) Immunohistochemical analysis of SRY, VASA, SOX9, AMH and DMRT1 in E34 fetal gonads. The cells were counterstained with hematoxylin. Scale bars in = 150 μm; in inset = 40 μm.

The *SRY* gene encodes a master protein for initiating testis differentiation in mammals. This gene was previously shown to be expressed during early gonad development in somatic cells of XY genital ridges and to induce supporting cell precursors to differentiate into Sertoli cells rather than female granulosa cells. Without normal Sry expression, gonads develop as ovaries or ovotestes^15^. We suspected that the abnormal promoter and gene body hypermethylation of the SRY gene might also occur in gonads and cause its abnormal expression, thus disrupting the highly regulated process of sex determination and leading to DSDs of XY^DSD^ dog. To confirm this hypothesis, we produced XY^DSD^ fetus at E34 by recloning of GSF74 (Supplemental Fig. 6A–E). Genetic sex in each individual gonad was further confirmed by PCR analysis of *SRY* gene sequences (Supplemental Fig. 6F). The XY^DSD^ fetus displayed a negative immunostaining signal for VASA, which is a marker of germ cells, whereas the signals were strongly positive in age-matched XX or XY fetuses produced by natural mating (Supplemental Fig. 7). We then compared methylation of the promoter of their SRY genes. The SRY promoter was significantly hypermethylated (Fig. 3b), while its mRNA expression was significantly reduced (Fig. 4a) in XY^DSD^ gonads relative to the XY^wt^ gonad at E34. These findings confirmed that SRY transcription was repressed by promoter hypermethylation. Furthermore, the *Sry* protein levels decreased in the XY^DSD^ gonads at E34, though it was not significantly reduced at this stage (Fig. 4b).

Despite the finding that *Sry* protein expression was only slightly reduced in the XY^DSD^ gonad at E34, we assumed that abnormal expression of *Sry* might still trigger a cascade of gene expression changes in the sex determination pathway. We next examined the expression levels of known fetal sex determining and maintenance genes^2^ that show sexually dimorphic expression between the XX^wt^ and XY^wt^ gonad. We found that, among testis-determining genes, mRNA expression of SF1, SOX8, SOX9, AMH and DMRT1 was significantly reduced in the XY DSD gonad compared to normal XY male gonads (Fig. 4c). Correspondingly, the proteins encoded by these genes were also clearly reduced (Fig. 4b). As these testis-determining genes are in the SRY downstream pathway of testis determination and differentiation, their repression was most likely correlated with down-regulation of the SRY gene. Most ovarian determining genes were expressed in low levels without showing significantly dimorphic expression patterns, except for the mRNA expression of CYP19A1, which was significantly up-regulated. Additionally, ERα was expressed in low levels in the XY DSD gonad (Fig. 4c). These results suggest that disruption of SRY gene expression led to a cascade reaction of expression changes in key genes involved in sex determination, with the testis determining genes being most repressed. In this early stage, highly up-regulated expression of CYP19A1 might indicate increased activity of estrogen biosynthesis, which can lead to subsequent female phenotypic sex development in these XY DSD dogs.

### Persistent SRY hypermethylation and enhanced expression of ovarian maintenance genes in postnatal XY DSD dogs

**Figure 5:**
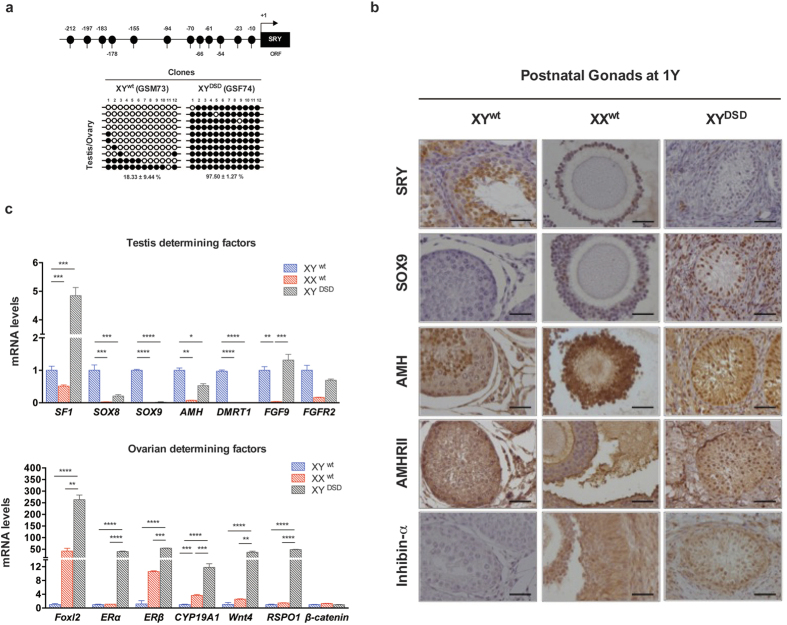
SRY methylation and gene expression of sex-determining genes in 1 year postnatal fetal gonads of XY^wt^, XX^wt^, and XY^DSD^ cloned dogs. (**a**) Promoter methylation status of SRY gene at 1 year postnatal gonad in the XY^wt^ and XY^DSD^ cloned dogs, using Bisulfite Sequencing PCR (BSP). (**c**) mRNA Expression of testis or ovarian determining factors were examined by QPCR. Significance of expression changes is indicated (one-way ANOVA). (**b**) Immunohistochemical analysis of SRY, SOX9, AMH, AMHRII and Inhibitinα. The cells were counterstained with hematoxylin. Scale bars in = 150 μm.

We also analyzed the *SRY* methylation pattern and expression of sex maintenance genes in normal XY testes, XX ovaries, and sex-reversal XY ovotestes at 1 year postnatal. Our methylation data of the postnatal gonads revealed that, once established, the SRY hypermethylation patterns were maintained during postnatal development of sex-reversal XY ovotestis (Fig. 5a). Similarly, mRNA and protein expression of all testis determining genes, including SF1, SOX8, SOX9, AMH and DMRT1, remained repressed for both XY DSD dogs (Fig. 5b,c). In contrast, the ovarian determining genes, including FOXL2, ERα, ERβ, CYP19A1, WNT4, and RSPO1, all showed highly up-regulated mRNA expression in XY DSD dogs, to a significantly higher level relative to normal XX ovaries (Fig. 5c). These results indicated that, due to abnormal hypermethylation and repressed expression of SRY, the equilibrium between testis and ovarian developmental program was disrupted. Once the developmental program was changed from male to female, it was maintained in postnatal life if no interference or treatment was provided.

## Discussion

Individuals with 46, XY complete gonadal dysgenesis are phenotypically female, have completely undeveloped streak gonads, and are often not diagnosed until puberty, when secondary sexual characteristics fail to develop. However, only a small number of patients can be explained by mutations in sex-determining genes^16^. By mediating the effects from environmental factors on genotype, epigenetics provides an alternative mechanism to explain 46, XY complete gonadal dysgenesis. In our DSD model of canine SCNT, we thoroughly screened the whole-genome and whole-methylome for potential anomalies of mutations or epimutations. Based on our large-scale genomic data, we managed to assemble canine Y chromosome sequences, which are absent from the previously published dog reference genome^9^. The new Y chromosome sequences generated in present study will provide a valuable tool for future investigations of the male dog genome. Despite a thorough examination of DSD dog genomes accompanied with comprehensive technological validation on candidate loci, our results revealed no potential genomic variations during SCNT that could explain the pathogenesis of DSD.

Conversely, because normal mammalian development involves complex regulation of active demethylation of gametes and *de novo* methylation of embryos after preimplantion^17^, accurate epigenomic reprogramming is also prerequisite for the success of SCNT^18^. However, extensive but stochastic anomalies of genome-wide DNA methylation were discovered in these SCNT DSD dogs. Most relevantly, persistent abnormal hypermethylation of the SRY gene, together with its down-regulated mRNA and protein expression, was observed. The *SRY* gene encodes a master protein for initiating testis differentiation in mammals. This gene is known to be expressed during early gonad development in somatic cells of XY genital ridges and to induce supporting cell precursors to differentiate as Sertoli cells rather than female granulosa cells. Previous studies indicated that the *SRY* gene can be expressed in Sertoli cells at different stages of fetal development until adult life^19^, as well as in a broad range of adult tissues in humans^20^,^21^. Experiments in mice showed that SRY expression level has to reach a certain threshold^22^ or have the correct timing^23^ to induce testis development. Once activated, Sry is assumed to first synergize with SF-1 to upregulate SOX9 expression^24^,^25^, which is a key hub gene for testis development that can stimulate expression of other downstream genes required for testis formation, such as AMH, FGF9 and FGFR2. In our study on XY DSD dogs, we found that the failure of SRY expression due to hypermethylation was well correlated with silencing of SOX9, and therefore also with a serial of testis determining genes, including, SF1, SOX8, AMH and DMRT1, in the early embryonic developmental stage in the XY^DSD^ gonad at E34 (Fig. 4). Accordingly, the results of our current study strongly indicate that incomplete demethylation of the *SRY* gene is the driving cause of XY^DSD^ in these SCNT cloned dog.

Our results also provided clear information regarding how failure of *SRY* expression triggered female development program during embryonic and fetal development due to antagonism between the testis- and ovary-determining regulatory networks. For instance, it was previously known that SOX9 can suppress the expression of ovarian genes such as RSPO1^26^ and FOXL2 in the XX genital ridge. DMRT1 can inhibit FOXL2 and prevent female reprogramming in the postnatal mammalian testis^27^, while FOXL2 also represses DMRT1^28^. Conversely, the silencing of these key testis-determination genes can lead to the activation of ovarian specific genes. Ovarian determining genes were mostly inactivated in the relatively early development stage of E34, except for CYP19A1, which encodes an enzyme critical for estrogen synthesis. However, we found that female specific genes were highly activated in the ovotestis after one postnatal year, including FOXL2, RSPO1, CYP19A1, Wnt4, ERα and ERβ (Fig. 5). Therefore, our results clearly indicated the central role of epigenetic regulation on *SRY* in sex determination and the fact that sex maintenance is more dynamic than previously acknowledged considering the dynamic nature of DNA methylation.

We also need to emphasize that our current study is about a special occasion of DSD that occurs during SCNT. As it mainly originated from the reprogramming procedure of SCNT, which is supposed to be a process of creating a genetically identical animal to the donor animal while epigenetically reprogrammed, the SCNT-induced DSD thereby is more prone to be epigenetically aberrant. However, the etiology of natural cases of DSD might vary from case to case. How frequent DSD in natural cases occurs due to epigenetic aberrancy remains an open question and needs to be further studied in the future.

## Methods

### Somatic cell nuclear transfer

All experimental protocols were performed in accordance with the Guidelines for the Care and Use of Laboratory Animals which approved by the animal ethics committee of the Sooam Biotech Research Foundation approved (Accession No: C-12-10). Both the oocyte donors and surrogates were mixed breed bitches aged 1 to 7 years with a body weight of 20–25 kg. The oestrus of the bitches was followed weekly by observing vulval bleeding, which signals the onset of the heat period. When in heat, a blood sample (2 mL) was collected once a day by cephalic venepuncture, and serum progesterone levels were measured by electro-chemiluminescence immunoassay (cobas e411, Roche Diagnostics). For oocyte retrieval, one side of the uterus horn was retracted and fixed with Allis forceps throughout the surgical procedure. The oviduct was flushed upward with TCM 199 (Life Technologies). This procedure was then repeated on the other side of the uterus horn. The surrounding cumulus cells from freshly retrieved oocytes were removed with 0.1% hyaluronidase. The SCNT procedure was performed as described previously^29^, with minor modifications. Before enucleation, the denuded MII oocytes were stained with 5 μg/ml Hoechst 33342 for 10 min. The polar bodies and metaphase chromosomes were aspirated, and the donor cells were subsequently inserted into the perivitelline space of the enucleated oocyte under an inverted microscope equipped with epifluorescence (TE2000-E, Nikon, Japan). A BTX Electro-Cell Manipulator 2001 (BTX, Holliston, MA, USA) was used to induce membrane fusion via application of two direct current (DC) pulses of 1.75 kV/cm for 15 μsec in 260 mM mannitol that contained 0.5 mM HEPES, 0.1 mM CaCl_2_ and 0.1 mM MgSO_4_. The reconstructed oocytes were cultured in TCM-199 at 39 °C under 5% CO_2_ until embryo transfer. Recipients were anesthetized as described earlier in oocyte retrieval and placed in ventral recumbency. The embryos were loaded into a tomcat catheter (Severeign, Sherwood Medical, MO, USA) with minimum medium volume, then gently transferred into the 2/3 distal position of the oviduct without insufflating air. Surrogates were checked for pregnancy by transabdominal ultrasound examination on Day 25 to 30 after embryo transfer^30^.

### Phenotypic analyses on XY^DSD^ dogs

#### Chromosomal analysis

After being cultured with 25 ng/ml colcemid for 4–6 h, cells were collected by trypsinization, treated with 0.075 M KCl for 15 min at 37 °C, and then fixed in methanol: acetic acid (3:1, v/v) solution. The cell suspensions were spread onto clean glass slides and air dried, after which the slides were stained with 5% Giemsa solution (Merck, Darmstadt, Germany) and metaphases were karyotyped using an Ikaros karyotyping system (Cal Zeiss, Jena, Germany).

#### Histological analyses

Ovary, uterine horn and uterine tube samples were collected from a 78 XY dog under general anesthesia. Immediately after collection of each sample, tissue pieces were processed for paraffin embedding after fixation in 10% buffered formalin. Each tissue was sectioned (5 μm), deparaffinized in xylene, and rehydrated through a graded ethanol series to distilled water before staining with hematoxylin and eosin (H & E).

#### GnRH stimulation test

To measure the plasma 17β-estradiol, progesterone, and testosterone concentrations, the GnRH analogue buserelin (loading dose: 0.4 μg/kg) was injected into the cephalic vein (Receptal^®^; Intervet SP). Blood samples were collected at 40 and 0 min before, and 10, 60, 90, and 120 min after its administration. Baseline testosterone levels were <1 ng/ml with little response to GnRH.

### Whole genome sequencing and analyses

#### NGS library construction and sequencing

Genomic DNA was extracted using a G-DEX™ IIc Genomic DNA Extraction Kit (iNtRON Biotechnology, Sungnam, South Korea). The WGS library construction followed a previously described protocol^31^. Approximately 1500 nanograms of genomic DNA in 100 μl TE buffer were fragmented to ~500 bp by the Covaris S2 system using the following parameters: cycle number, 1; duty/cycle, 20%; intensity, 5; cycles/burst, 200; and time, 20s. The 3′ or 5′ overhangs of the fragmented DNA were converted to blunt ends using T4 DNA polymerase, T4 Polynucleotide Kinase and Klenow polymerase for 30 min at 20 °C, then purified using the column of a QIAquick PCR Purification Kit (Qiagen). Purified DNA was subsequently A-tailed with dATP and Klenow (3′-5′ exo-) for 30 min at 37 °C, then purified with the Mini column of a MiniElute PCR Purification Kit (Qiagen). Next, DNA adaptors (Illumina) with a single “T” base overhang at the 3′ end were ligated to the above products with T4 DNA ligase at 20 °C for 15 min, then purified with the column of a QIAquick PCR Purification Kit (Qiagen). The purified products were subsequently separated on a 2% agarose gel, excised from the gel at a position between 580 and 600 bp, and purified (Qiagen Gel Extraction Kit). The adaptor-modified DNA fragments were enriched by PCR with PCR primer 1.1 and Index N primer 2.0 (Illumina) for eight cycles using Phusion DNA Polymerase (New England Bioloabs). Deep WGS was then performed using the Illumina HiSeq2000 platform (500 bp library, 90 bp reads).

#### Alignment of sequencing reads and assembly of Y chromosome

Low-quality reads, i.e., reads with more than 50% low quality base (Q-score < 20) or with more than 10% N base, were first removed from the raw sequencing reads. The “rmdup” module interpolated in Samtools was used to remove PCR duplicates. The SOAP (version2.01) or BWA algorithms were then applied to map the clean reads to the reference boxer genome (canFam2.0) from the Ensembl database (ftp://ftp.ensembl.org/pub/release-66)^9^. Following alignment, we took all un-aligned pair-end reads from all samples as the candidate reads that originated from chromosome Y and used SOAPdenovo assembler (ver.1.06) to build a *de novo* draft assembly for dog chromosome Y. The low-frequency k-mers regions were corrected or removed. All reads that were uniquely mapped to the genome were used in the subsequent analyses.

#### Detection of SNVs and indels

VarScan (v2.2.11)^32^,^33^ was used to compare the clone sample with the donor sample to search for potential SNVs and indels, for which several heuristic rules were applied for a specific genomic locus: (1) the base quality should be no less than 20 for more than 50% of the bases, while the N-base should be less than 10% in each read of both the donor and matched clone samples; (2) both the clone and matched donor samples should be covered at depths of ≥5x and ≤100x; (3) the variant frequency (supportive read for variant/sum of all reads) should be larger than 0.2 for somatic mutations and larger than 0.75 or less than 0.25 for LOH in clone samples; and (4) the variants should be supported by at least two reads in the clone samples; (5) the GATK software was applied independently to call somatic mutations and LOHs^34^,^35^, after which the results were integrated with VarScan by reasoning that true somatic mutations and LOHs should be covered in both results. We then searched these potential somatic mutations and LOHs in the ENSEMBL and dbSNP databases (ver.CanFam2.0) to eliminate the known somatic mutations and LOHs that were previously described as germline variants and filtered out the variants located in repeated regions of the dog genome.

#### Detection of structural variation

Three algorithms, CNVnator, which performs read-depth (RD) analysis^36^, Pindel, which detects split reads (SP) or break points^37^ and Breakdancer, which detects anomalous read pairs (RP)^38^, were integrated for identification of potential structure variations (SV). Six types of SVs were analyzed, deletions (DEL), tandem duplications (TD), insertions (INS), inversions (INV), intra-chromosomal translocations (ITX) and inter-chromosomal translocations (CTX). For DEL, TD and INS, medium or large SVs were defined to support the three algorithms. The parameters of calling each type of SV were adjusted according to developer’s instructions. A union of the results from the three algorithms was created to build a set of particular SVs.

### Whole-methylome sequencing and analyses

#### NGS library construction and sequencing

Deep WGBS were performed using the Illumina HiSeq2000 platform (500 bp library, 90 bp reads) as previously described, with minor changes^39^. Approximately 1500 nanograms of genomic DNA in 100 μl TE buffer were fragmented to ~200–300 bp by the Covaris S2 system using the following parameters: cycle number, 3; duty/cycle, 10%; intensity, 5; cycles/burst, 200; and time, 40 s. Each sequencing library was constructed similarly to genomic DNA libraries, except that ligation was performed using methylated adapters provided by Illumina. Ligation products were purified using a Mini column from a MiniElute PCR Purification Kit, after which the ligated DNA was treated with bisulfite using the EZ DNA Methylation-Gold Kit™ (ZYMO). The purified products were subsequently separated on a 2% agarose gel, excised from the gel at a position between 320 bp and 420 bp, and purified (Qiagen Gel Extraction Kit). The products were then enriched by PCR with PCR primer 1.1 and Index N primer 2.0 (Illumina). PCR was conducted using JumpStart™ Taq DNA Polymerase (Sigma) and the following PCR conditions: 30s at 94 °C, followed by 10 cycles of 30 s at 94 °C, 30 s at 60 °C, 30s at 72 °C and then final extension for 5 min at 72 °C.

#### Data analyses

The SOAP2 algorithm^40^ was applied to map the sequencing reads back tothe reference boxer genome (canFam2.0). The methylation level of CpG sites was calculated as N_me_/(N_me_ + N_non-me_), where N_me_ represents the methylation-supportive read number and N_non-me_ represents the non-methylation-supportive read number. “Pvclust” was performed for hierarchical clustering analysis^41^. CpG island information and other repeat region information were obtained from the UCSC database (ftp://hgdownload.cse.ucsc.edu/goldenPath/canFam2/database/). Putative DMRs were then identified by comparison of two methylomes using the strategy of sliding windows^39^, which mainly consisted of the following steps: first, a CpG site with significantly different methylation was selected as the start site for DMR (CpG depth in donor and clone > = 4, Fisher test p value ≤ 0.01 and absolute value of methylation level differences ≥0.2). Second, we added the downstream adjacent CpG sites to the initial DMR. The DMR extension had to meet several criteria: (1) CpG depth in donor and clone ≥4; (2) Fisher test p value ≤ 0.01; (3) the methylation tendency (differences in methylation level between donor and clone samples) of the added CpG sites could be opposite to the start site of DMR, but the Fisher test p value was >0.01 (single CpG site); (4) the methylation tendency of two adjacent CpG sites is not opposite to that of the start site of DMR; (5) the distance of two adjacent CpG sites ≤200 bp; and (6) DMR length ≤2000 bp[ED highlight-same comment as above.]. Third, we removed the insignificant CpG sites (Fisher test p value > 0.01) backwards until we encountered a significant CpG site (Fisher test p value ≤ 0.01) to eliminate the obscure boundary of the DMR. Finally, we filtered all DMR regions according to the following standards: (1) CpG sites number ≥5; (2) methylation level difference in DMR ≥0.2; and (3) DMR length ≥200.

### MiSeq amplicon sequencing

For SNPs and indels located in exonic regions, Illumina MiSeq amplicon sequencing was applied on the barcoded PCR products from different samples. PCR primers that uniquely amplify 26 exonic variations and exonic regions of 8 sex determination genes were designed by Primer Premier 5.00 (Premier Biosoft), and length of all PCR products was controlled around 450 bp (Supplemental Table 6). For each sample, genomic DNA, KAPA2G Robust HotStart ReadyMix (KAPA Biosystems), PCR primers, PCR primers mediated high throughput sequencing linkers with a barcode sequence were dispensed into one individual nanowell of a Smart MyDesign Chip, which contains 5184 nanowells, by Smart Chip Multisample Nanodispenser. 36 amplicons for each sample and 150 samples could be constructed as one library with barcode sequence classifying samples using one Smart MyDesign Chip. PCR amplification of Smart MyDesign Chip was conducted using the Techne Prime Thermal Cycler. PCR product was purified using the QIAquick Gel Extraction Kit (Qiagen). After analyzed by an Agilent 2100 Bioanalyzer (Agilent Technologies) and quantified by real time PCR, the library was sequenced with pair end 250 bp using Illumina Miseq sequencer. When sequencing finished, the Illumina reads were post-processed and aligned to the reference regions (all PCR regions). SNVs and indels were detected with the same procedure described above (Supplemental Table 7).

### Bisulfite sequencing PCR

BSP was conducted using an EZ DNA Methylation-Gold™ Kit according to the manufacturer’s protocols. The process started with amplification of the bisulfite-treated SRY downstream promoter containing 12 CpG sites using sequential PCR reactions from bisulfite-treated DNA with the following nested primers: methyl_SRY-F: 5′-GTT AGA TGT TGA TTT TAT TTG GGA-3′ and 5′-AAT CAT CGC AAT TCA ATA CCC-3′. PCR was performed on genomic DNA using ZymoTaq™ DNA polymerase according to the manufacturer’s instructions (Zymo Research). A total of 100 ng of bisulfite-treated genomic DNA was used as the template for PCR, and the PCR cycles were as follows: 95 °C for 10 min, followed by 40 cycles of 96 °C for 30 sec, 60 °C for 30 sec, and 72 °C for 30 sec, and then final extension at 72 °C for 7 min. The PCR products were purified by gel extraction from a 2% agarose gel and ligated into the pGEM-Teasy vector (Promega), after which the ligation products were used to transform competent *Escherichia coli* cells (strain DH5a) using standard procedures. Blue/white screening was then used to select a minimum of 20 bacterial transformants (clones). Plasmid DNA was subsequently isolated from each clone using a Miniprep Kit (Solgent). Next, clones were screened by agarose gel electrophoresis to verify the insert and plasmid size. Sequencing of the SRY promoters of the positive clones was then conducted by Solgent sequencing service (Solgent, Daejeon, South Korea), after which the sequences were compared with the original sequences. The sequenced clones with complete sequencing data and a minimum of 95% bisulfite conversion were included in subsequent analyses.

### Quantitative PCR

Total RNA from either fetal or postnatal gonads was extracted using TRizol reagent^TM^ (Invitrogen) and an RNeasy mini kit (QIAGEN). cDNA was then synthesized using High-Capacity cDNA Reverse Transcription Kits (Applied Biosystems) and analyzed by quantitative PCR (Q-PCR) using a Power SYBR Green PCR master mix (Applied Biosystems) and the ABI 7300 Real-Time PCR System (Applied Biosystems). All data were normalized to ribosomal GAPDH expression. Relative transcription levels between the sexes for each type of XY male, XX female, and XY DSD cloned dog were examined by analysis of variance (ANOVA) and a Newman-Keuls multiple comparison tests. All data are expressed as the mean ± SEM, and a probability of P < 0.05 was considered statistically significant in all tests.

### Data Access

WGS data were deposited in the Sequence Read Archive (SRA) with accession of SRA304271. WGBS data were deposited in Gene Expression Omnibus (GEO) with accession of GSE74225. The newly assembled Y chromosome reference sequences were deposited in NCBI Whole Genome Shotgun (WGS) database (accession ID: PRJNA305243).

## Additional Information

**How to cite this article**: Jeong, Y.-H. *et al*. Stochastic anomaly of methylome but persistent SRY hypermethylation in disorder of sex development in canine somatic cell nuclear transfer. *Sci. Rep.* **6**, 31088; doi: 10.1038/srep31088 (2016).

## Supplementary Material

Supplementary Dataset 1

Supplementary Dataset 2
