# Supplementary material for: Stochastic anomaly of methylome but persistent SRY hypermethylation in disorder of sex development in canine somatic cell nuclear transfer
Source: Sci Rep. 2016 Aug 9;6:31088. doi: 10.1038/srep31088 (PMC4977463; doi:10.1038/srep31088)
Supplement: Supplementary Dataset 1 [file srep31088-s1.doc]

# Stochastic anomaly of methylome but persistent SRY hypermethylation in disorder of sex development in canine somatic cell nuclear transfer

Young-Hee Jeong^1#^, Hanlin Lu^2,3#^, Chi-Hun Park^1,4,5^, Meiyan Li^2^, Huijuan Luo^2^, Joung Joo Kim^1^, Siyang Liu^2^, Kyeong Hee Ko^1^, Shujia Huang^2^, In Sung Hwang^1^, Mi Na Kang^1^, Desheng Gong^2,3^, Kang Bae Park^1^, Eun Ji Choi^1^, Jung Hyun Park^1^, Yeon Woo Jeong^1^, Changjong Moon^6^, Sang-Hwan Hyun^1,7^, Nam Hyung Kim^8^, Eui-Bae Jeung^7^, Huanming Yang^2^, Woo Suk Hwang^1*^, Fei Gao^2,3*^

**Affiliations**

^1^ Sooam Biotech Research Foundation, Seoul 152-904, Korea.

^2^ BGI-Shenzhen, Shenzhen, China

^3^ Agricultural Genomics Institute at Shenzhen, Chinese Academy of Agricultural Sciences, Shenzhen, China

^4^ Animal Bioscience and Biotechnology Laboratory, United States Department of Agriculture, Beltsville, MD, 20705, USA

^5^ Department of Animal and Avian Sciences, University of Maryland, College Park, MD, 20742, USA

^6^ College of Veterinary Medicine, Chonnam National University, Gwangju 500-757, Korea

^7^ College of Veterinary Medicine, ^8^ College of Animal Sciences, Chungbuk National University, Cheongju, Chungbuk 361-763, Korea

# These authors contributed equally to this work.

* **Corresponding author:**

FG: [flys828@gmail.com](mailto:flys828@gmail.com)

BGI-Shenzhen & Agricultural Genomics Institute at Shenzhen,

7 Pengfei, Dapeng New District, Shenzhen, China, 518120

Tel: 86-755-23251432

WSH: hwangws@sooam.org

Sooam Biotech Research Foundation

64 Kyunginro, Guro-gu, Seoul, South Korea, 152-100

Tel : 82-2-2616-5658

Fax : 82-2-2616-5672

**Running title**: *SRY* hypermethylation in XY^DSD^ SCNT dogs

## Supplemental figure

**
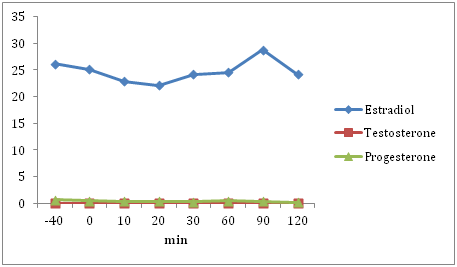
**

**Supplemental Figure 1. Anomaly of hormones levels (testosterone, estradiol and progesterone) in** **XY^DSD^ dogs.**


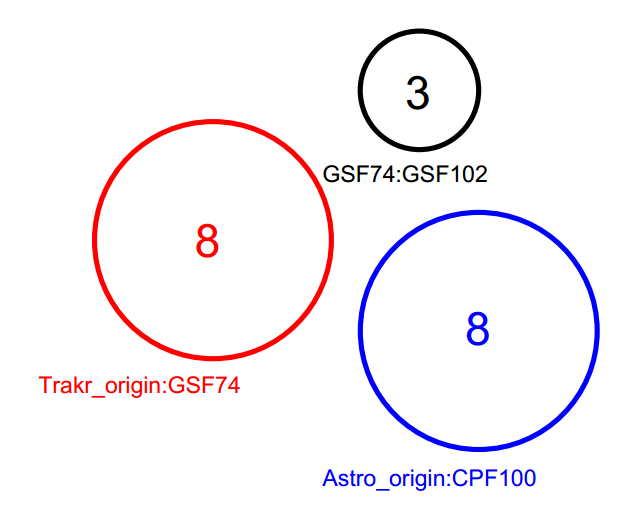


**Supplemental Figure 2. Recapitulated exonic variants display no overlap among the three clone-control pairs.**


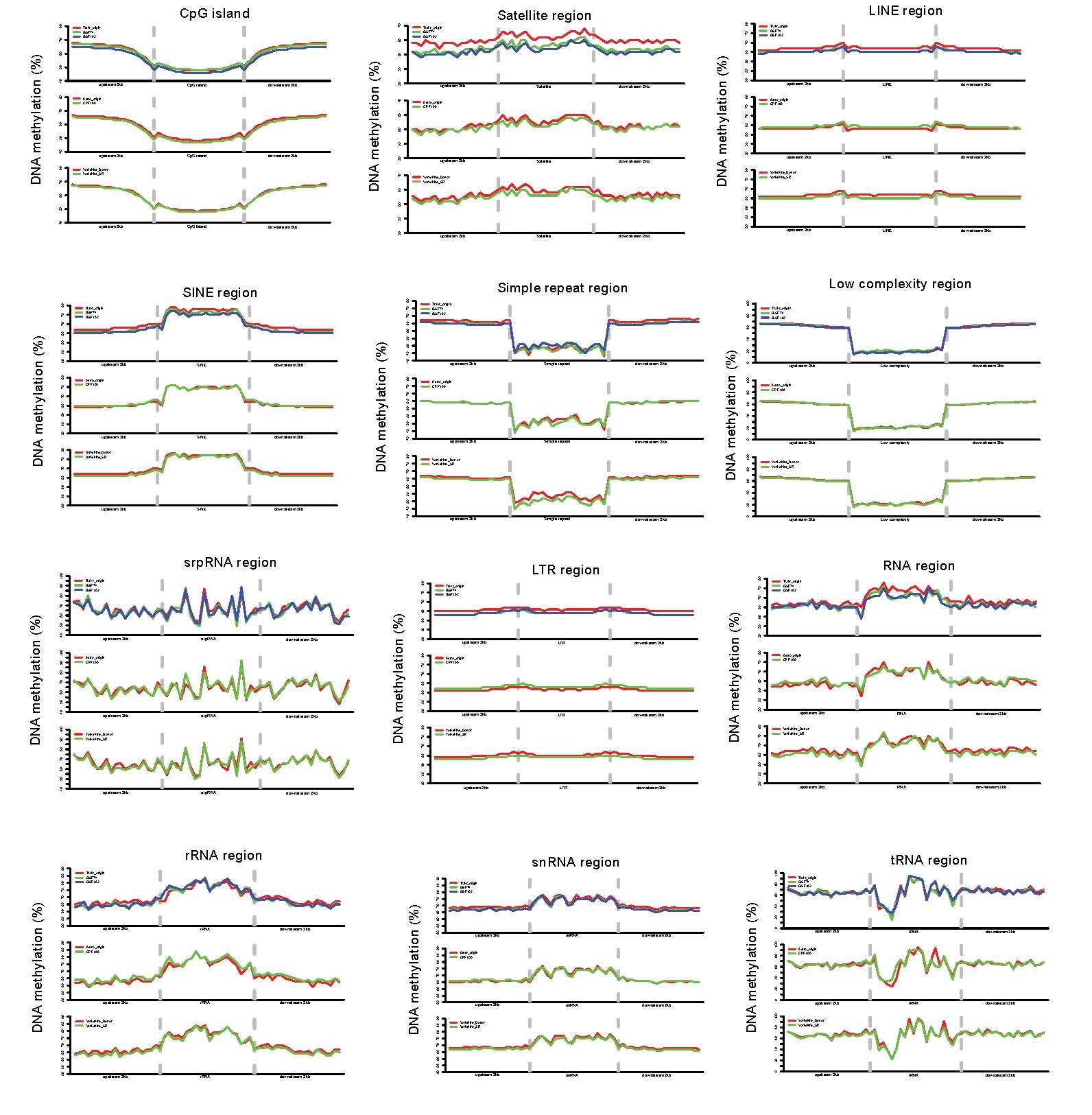


**Supplemental Figure 3. Average methylation levels of CpG sites display no significant difference in each genomic element.** Two-kilobase upstream and downstream of each genomic element were divided into 100–bp intervals. Each gene was divided into 20 intervals (5% per interval). Plots show the Average methylation levels of each interval.


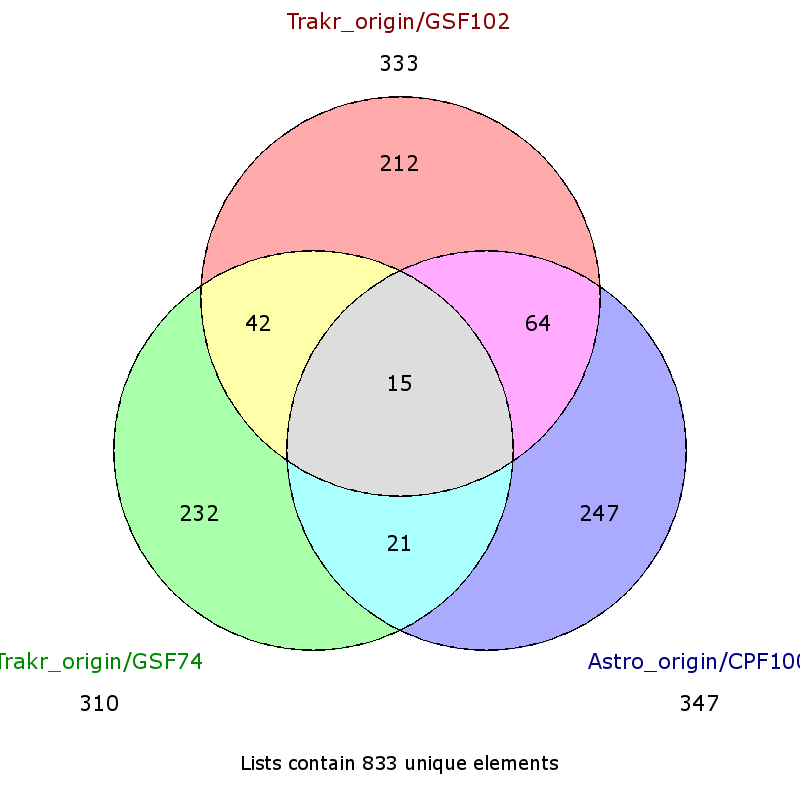


**Supplemental Figure 4. DMR-containing genes were not enriched in** **process of sex-reversal.** Venn diagram showed the overlap of DMR-containing genes between three pairs of donor-clone comparisons.


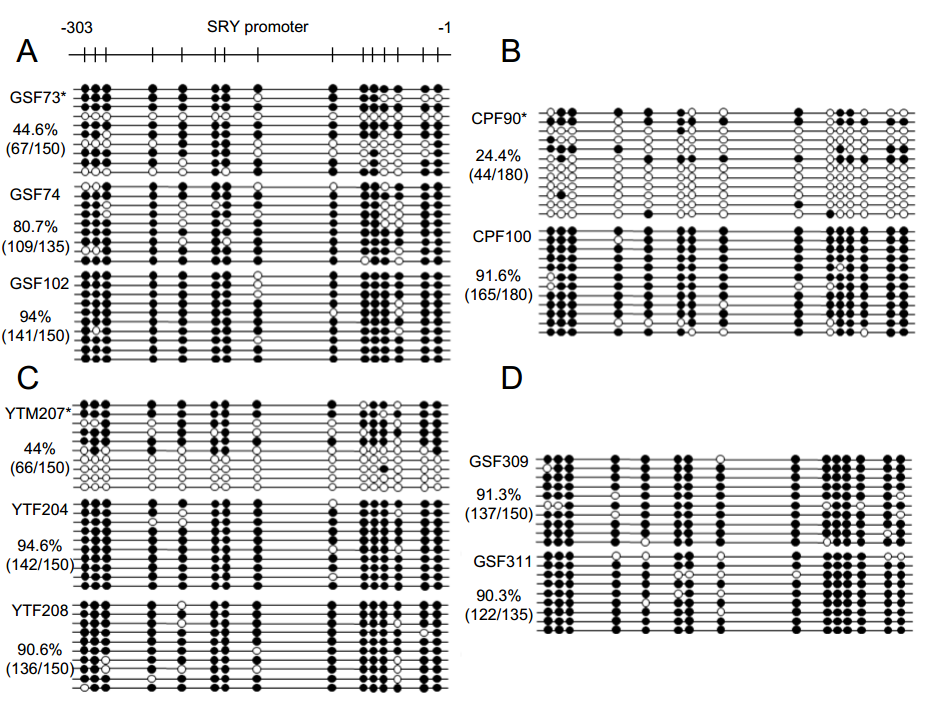


**Supplemental Figure 5. Methylation validation of SRY promoter in cloned dogs with or without DSD.** Results from cloned dog offsprings that were produced from donors of Trakr-origin clone (A), Astro-origin clone (B), Yorkshire Terrier-origin clone (C) and 'Raccoons'-origin clone (D) are presented. The data are generated using bisulfite sequencing PCR technology. 10-15 sequences were randomly picked for each sample. Open circles represent unmethylated CpGs, while filled circles represent methylated CpGs. * stands for cloned dogs with normal sexual characteristics, while the others are sex-reversed cloned dogs.


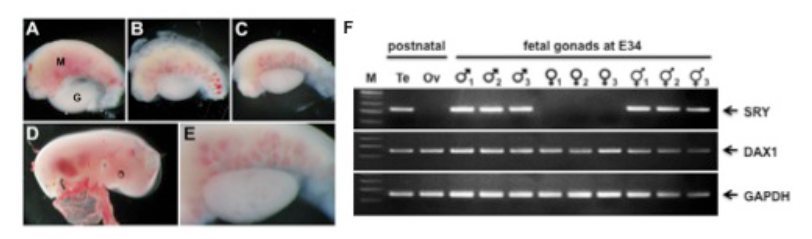


**Supplemental Figure 6. Normal Sry expression reoccurred in recloning XY^DSD^ fetus.**


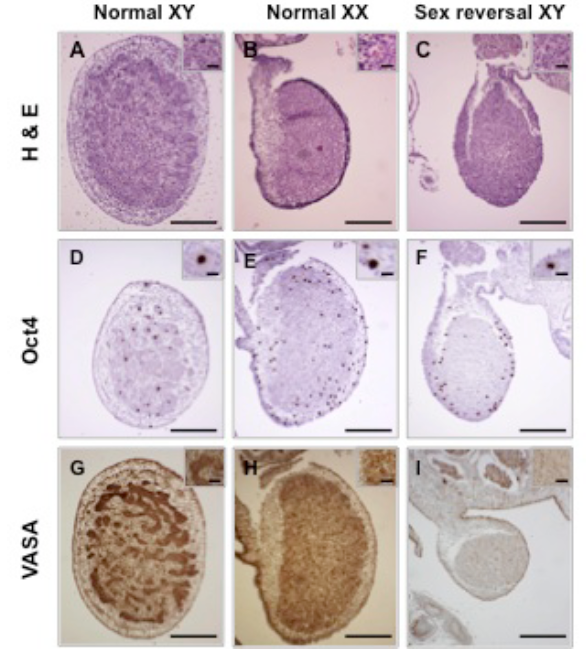


**Supplemental Figure 7. Silence of VASA in** **recloning XY^DSD^ fetus.**
